# Reviewing the risk of feed as a vehicle for swine pathogen transmission

**DOI:** 10.1002/vms3.227

**Published:** 2019-12-18

**Authors:** Cassandra K. Jones, Jason Woodworth, Steve S. Dritz, Chad B. Paulk

**Affiliations:** ^1^ Department of Animal Sciences and Industry Kansas State University Manhattan KS USA; ^2^ Department of Diagnostic Medicine/Pathobiology Kansas State University Manhattan KS USA; ^3^ Department of Grain Science and Industry Kansas State University Manhattan KS USA

**Keywords:** animal food, bacteria, biological, feed, hazard, virus

## Abstract

**Background:**

While porcine biological hazards have had the potential to be transmitted through feed and feed mills for decades, the emerging threat of foreign animal disease has elevated the concern that these may enter or be transmitted throughout the domestic swine herd via a feed vehicle.

**Objective:**

The goal of this review was to describe the current classification for emerging porcine biological pathogen transmission through the feed supply chain so resources can be best directed towards those of highest risk.

**Methods:**

By assessing the pathogen severity to pigs and the probability of pathogen transmission through feed, an overall risk can be established using a hazard analysis matrix.

**Results:**

There is negligible risk for feed‐based transmission of a transmissible spongiform encephalopathy, *Trichinella spiralis*, *Toxoplasma gondii*, *Salmonella* Choleraesuis, *Salmonella* spp. except Choleraesuis and I 4,[5],12:i:‐, porcine deltacoronavirus, Senecavirus A, mammalian orthoreovirus 3, foot and mouth disease virus, classical swine fever virus or Chinese pseudorabies virus. However, the combined severity and probability of *Salmonella enterica* serotype I 4,[5],12:i:‐, porcine epidemic diarrhoea virus and African swine fever virus warrant a moderate risk characterization for transmission through the US feed supply chain.

**Conclusions:**

This risk can be maintained below critical status by minimizing the likelihood that a pathogen can enter the feed supply chain, such as by excluding high‐risk ingredients from facilities, extending biosecurity to mills, and considering proactive mitigation strategies. In reality, all these actions may be necessary to prevent the detrimental transmission of porcine biological hazards into the US swine herd through the feed supply chain.

## Graphical abstract

While porcine biological hazards have had the potential to be transmitted through feed and feed mills for decades, the emerging threat of foreign animal disease has elevated the concern that these may enter or be transmitted throughout the domestic swine herd via a feed vehicle. By assessing the pathogen severity to pigs and the probability of pathogen transmission through feed, an overall risk can be established using a hazard analysis matrix. This risk can be maintained below critical status by minimizing the likelihood that a pathogen can enter the feed supply chain, such as by excluding high‐risk ingredients from facilities, extending biosecurity to mills and considering proactive mitigation strategies.

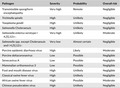

## 1 INTRODUCTION

The US swine industry has made substantial gains in herd health by implementing farm biosecurity practices. Many of the primary routes of pathogen entry into the farm (i.e. other pigs, farm employees, visitors, air, etc.) have been minimized in high health systems, whereas less research has characterized the risk of feed as a vector of disease transmission. Much of the research thus far has focused on pathogens that are domestic threats in the United States, such as porcine epidemic diarrhoea virus (PEDV) or *Salmonella*. As foreign animal diseases, such as African swine fever virus (ASFV), classical swine fever virus (CSFV) and foot and mouth disease, circulate among a number of global trade partners, the concern for ingredients as a vector of transboundary disease entry has increased. While not all pathogens are strong candidates for feed‐based transmission, it is important to characterize the risk for the feed supply chain to serve as a vehicle for pathogen entry into farms. Research in this area is ongoing, so a review of current knowledge is important to form a foundation from which to strategically address research gaps. As such, our objective is to characterize pathogenic biological hazards that may be a risk for entering through the feed supply chain, and to describe their potential prevention or mitigation.

## 2 MATERIALS AND METHODS

### 2.1 Assessing the risk of swine pathogens in feed

In response to the Food Safety Modernization Act, the US Food and Drug Administration (FDA) published 21 CFR Part 507: Current Good Manufacturing Practice, Hazard Analysis and Risk‐Based Preventive Controls for Food for Animals. This rule requires facilities that manufacture animal food for consumption in the United States to conduct a hazard analysis of agents that may cause illness or injury to humans or animals via the animal food. The rule requires that the assessment of severity and probability of an agent be used to determine overall risk, which allows a system to focus its resources on the most critical areas. This review relies on the same required methodology. In the case of pathogens in feed, the severity of a hazard is typically consistent across production systems, feed mills and even countries. However, the assessment of hazard probability is highly variable, depending upon a wide variety of circumstances that are unique to each system. For example some pathogens, such as *Salmonella*, have the ability to impact both human and animal health, whereas others, such as PEDV, have no impact on human illness. Furthermore, the type of raw materials included in a diet, the environment in which feed is manufactured, the transportation of ingredients and feed to and from the facility, the prerequisite programs enforced and the training of the individuals involved in each process can all change the probability of hazard occurrence. Therefore, the classification of severity, and especially probability, should be conducted by each individual system. It is the authors’ hope that this paper will facilitate that assessment, not replace it. For the purpose of this paper, assumptions include: (a) pathogen presence in the United States is as of September 1, 2019; (b) all entities manufacturing, processing, packing, holding, or transporting animal food are in compliance with all federal food safety regulations; (c) pigs are raised in indoor, conventional US commercial production.

### 2.2 Severity

The assessment of severity includes evaluating its ability to cause illness or injury to humans or animals, regardless of its probability. Classifications were determined as described in Table 1. Severity is associated with the pathogen itself, and is unrelated to its likelihood of contamination, survivability or infectivity. Its assessment is also indecent of systems placed for prevention or mitigation.

**Table 1:** Classification of severity assessment based on impact on human or animal health

Classification	Impact on human illness or injury	Impact on animal illness or injury
Very high	Potential death or serious illness without recovery in humans	—
High	Potential minor illness from which full recovery is likely or no possible impact on human health	Potential death of many animals
Moderate	No possible impact on human health	Potential death or serious illness without full recovery of many animals
Low	No possible impact on human health	Potential death or serious illness without full recovery of few animals or minor illness from which full recovery is likely in many of animals
Very Low	No possible impact on human health	Potential minor illness from which full recovery is likely of few animals

### 2.3 Probability

The assessment of probability includes evaluating its likelihood of containing the pathogen, regardless of its severity. Classifications were determined as described in Table 2. Probability is associated with the likelihood of contamination, survivability or infectivity of a pathogen. Its assessment is also impacted by systems placed for prevention or mitigation. However, probability is independent of the possible severity the pathogen may have on human or animal health.

**Table 2:** Classification of probability assessment based on likelihood of occurrence

Classification	History of occurrence	Likelihood of future occurrence
Almost certain	Is occurring currently	—
Likely	Has recently occurred	Probable to occur again
Possible	Has not recently occurred	Possible to occur in the next 6 months
Unlikely	Has not ever occurred	Unlikely to occur in the next 12 months
Remote	Has not ever occurred	Unprecedented and remotely likely to occur in next 36 months

### 2.4 Assessment of risk

To assess overall risk to the system, the severity and probability must then be considered collectively, as demonstrated in Table 3. It is the combination of severity and probability that impact overall risk of feed as a vehicle for swine pathogen transmission.

**Table 3:** Classification of risk based on the combination of severity and probability

Probability	Severity				
	Very high	High	Moderate	Low	Very low
Almost certain	Critical	Critical	Moderate	Moderate	Negligible
Likely	Critical	Moderate	Moderate	Negligible	Negligible
Possible	Moderate	Moderate	Negligible	Negligible	Negligible
Unlikely	Moderate	Negligible	Negligible	Negligible	Negligible
Remote	Negligible	Negligible	Negligible	Negligible	Negligible

## 3 RESULTS AND DISCUSSION

### 3.1 Pathogens of concern for feed‐based transmission

#### 3.1.1 Prions and parasites

Prior to 2010, the primary biological hazard of concern for US feed mills were transmissible spongiform encephalopathies (TSE) caused by prions, such as bovine spongiform encephalopathy. There is limited evidence of animal‐to‐human transmission and its impact on a large number of animals, leading to a very high severity. While TSE transmission to pigs has been established in experimental conditions, it is not known to occur naturally in swine (Hedman et al., 2016). Regulations established by the FDA in 1997 and 2008 have successfully controlled the domestic spread of the disease, which is recognized as the United States has negligible BSE risk according to the World Health Organization, and therefore the probability of a swine‐based TSE is remote (World Organisation for Animal Health, 2018). This leads to an overall risk ranking of negligible (Table 4).

**Table 4:** Assessment risk for feed to be a vehicle for swine pathogen transmission

Pathogen	Severity	Probability	Overall risk
Transmissible spongiform encephalopathy	Very high	Remote	Negligible
*Trichinella spiralis*	High	Unlikely	Negligible
*Toxoplasma gondii*	High	Unlikely	Negligible
*Salmonella* Choleraesuis	High	Unlikely	Negligible
*Salmonella enterica* serotype I 4,[5],12:i:‐.	Very high	Unlikely	Moderate
*Salmonella* spp. except Choleraesuis and I 4,[5],12:i:‐	Very low	Almost certain	Negligible
Porcine epidemic diarrhoea virus	High	Likely	Moderate
Porcine deltacoronavirus	Low	Possible	Negligible
Senecavirus A	Low	Possible	Negligible
Mammalian orthoreovirus 3	Low	Possible	Negligible
Foot and mouth disease virus	High	Unlikely	Negligible
Classical swine fever virus	High	Unlikely	Negligible
African swine fever virus	High	Possible	Moderate
Chinese pseudorabies virus	High	Unlikely	Negligible

Both *Trichinella spiralis* and *Toxoplasma gondii* have historically been linked to feed‐based transmission and have potential impact on human health, leading to its high severity (Guo et al., 2015; Slifko, Smith, & Rose, 2000). However, biosecurity improvements and indoor all‐in/all‐out management have largely eradicated the hazards from the domesticated swine heard (Davies, Morrow, Deen, Gamble, & Patton, 1998). In a study of North Carolina swine farms in 1998, just 1 of 2,175 (0.057%) pigs housed in confinement was seropositive for *T. gondii* and 1 of 2,183 (0.046%) pigs was positive for antibodies against *T. spiralis* (Davies et al., 1998). Even then, the resulting carcass processing and meat preparation must be inadequate for illness to result, leading to an unlikely probability. These data suggest that feed‐based transmission of prions or parasites to domestic pigs is a negligible risk (Table 4).

#### 3.1.2 Bacteria

While the poultry feed and pet food industries have evolved to control *Salmonella* spp., reports of pathogenic bacteria transmission from feed to swine have been limited. The FDA recognizes that swine feed is rarely in direct contact with immunocompromised people, and therefore the risk of salmonellosis in humans from swine feed is negligible. Furthermore, the FDA has determined that feed containing most *Salmonella* serotypes, such as *Salmonella* Enteritidis or *Salmonella* Typhimurium, are not likely to cause swine disease. For these reasons, *Salmonella* Choleraesuis is the only serotype considered by FDA to be an adulterant in swine feed (Food & Drug Administration, 2013). In its routine surveillance for *Salmonella* spp. in animal feeds and ingredients, 19.4% of ingredients and 5.6% of complete feeds contained the pathogen (Li et al., 2012). However, no *Salmonella* Choleraesuis was identified, causing FDA to conclude that it is unlikely for feed to cause salmonellosis in pigs. Based on this data, it is determined that *Salmonella* Choleraesuis transmission through feed would be a high severity, but unlikely probability, leading to negligible overall risk (Table 4).

Outside of the regulatory environment, there is still concern for other serotypes of *Salmonella* spp. In particular, there is rising concern with an emerging serotype, *Salmonella enterica* serotype 4,[5],12:i:‐ in swine. This is a potential monophasic variant of *Salmonella* Typhimurium, and highly resistant to multiple drugs, including ampicillin, streptomycin, sulfonamides and tetracyclines (Elnekave et al., 2018). The serotype is most commonly linked to pork products, and was responsible for a substantial recall of whole roaster hogs from the state of Washington, but was not been linked to feed (Elnekave et al., 2018). Recently, *S. enterica* serotype 4,[5],12:i:‐ has again been linked to swine; a recall of pig ears intended to be used as pet treats was linked to illness in at least 93 people in 27 states (CDC, 2019). A 2016–2017 study reported the presence of *S. enterica* serotype 4,[5],12:i:‐ in 12 US swine feed mills sampled from feed and mill surfaces over three different seasons (Magossi et al., 2019). Five of 696 feed mill environmental samples (0.72%) and 0 of 39 feed samples were identified to include the pathogen via RT‐PCR. Of the positive environmental samples, three were from dust collected on the floor of the bulk ingredient receiving area, one from the bulk ingredient receiving pit, and one from the control room floor. The five positive samples were from three mills located in North Carolina, Kansas and Indiana, with two positive samples recovered in Fall 2016 and three positive samples recovered in Summer 2017. Thus, this serotype has been found in the environment of US feed mills, but not in finished feed or on a direct feed‐contact surface. Based on the available data, it is concluded that *S. enterica* serotype 4,[5],12:i:‐poses very high severity and unlikely probability for occurrence, therefore having a moderate overall risk (Table 4). Additional research is needed to further assess the root cause and prevalence of *S. enterica* serotype 4,[5],12:i:‐.in the feed‐to‐pig‐to‐pork supply chain due to its significance on human health and presence in the environment of swine feed mills.

There are limited links of feed‐based transmission of other *Salmonella* serotypes resulting in animal or human health concerns. Across all samples from the study above, 8% of feed samples, 13% of bulk ingredient receiving pits, 11% of feed mill floors and 14% of worker shoes were positive for *Salmonella* spp. (Magossi et al., 2019). Further investigation determined that the isolates belonged to 15 different serotypes of *S. enterica*: Agona (14), Mbandaka (13), Seftenberg (7), Schwarzengrund (6), Rissen (3), Hartford (2) and Typhimurium (2) Bareilly (1), Braenderup (1), Cubana (1), Javiana (1), Kiambu (1), Poona (1), Soerenga (1), Worthington (1) and I 4,[5],12:i:− (1) (Lomonaco et al., 2018). Notably, *Salmonella* Choleraesuis was not identified. Based on limited data linking other *Salmonella* serotypes to animal health or resulting human illness, the severity of *Salmonella* spp. beyond *Salmonella* Choleraesuis and I 4,[5],12:i:‐ is characterized as very low, whereas the probability is almost certain, leading to an overall risk of negligible (Table 4). As the swine industry has continued pressure to eliminate sources of antibiotic‐resistant bacteria from pork, the feed‐to‐fork food safety will continue to be an emerging concern. It will become important to characterize the serotypes of *Salmonella* more fully and understand their source of entry and potential transmission to impact animal or human health.

#### 3.1.3 Viruses

The major swine‐based viruses with concern for feed‐based transmission do not impact humans. The swine industry first recognized the significant role that the feed supply chain can play in pathogen transfer in 2013–2014 with PEDV. This virus caused substantial mortality and morbidity in a large numbers of animals, leading to its severity assessment of high. In its 2015 report evaluating the root cause of PEDV, the US Department of Agriculture determined that ‘the use of Flexible Intermediate Bulk Containers (aka: FIBC or “tote bags”) best fit the criteria established for entry in to the United States, rapid and wide spread across the country, and introduction onto individual farms (US Department of Agriculture Animal & Plant Health Inspection Service Veterinary Services, 2015)’. Furthermore, feed or ingredients were linked to outbreaks of PEDV in both the United States and Canada (Aubry, Thompson, Pasma, Furness, & Tataryn, 2016; Pasick et al., 2014).Improvements in biosecurity have lessened these reports in recent years, but PEDV transmission through feed still carries a likely probability, for an overall risk of moderate (Table 4). If biosecurity procedures are breached or prevalence surges above the epidemiological threshold, feed‐based transmission may rise to a critical risk.

Other domestic porcine viruses, such as porcine deltacoronavirus, Senecavirus A and mammalian orthoreovirus 3 are low severity due to their likelihood to impact fewer animals with less fatal consequences. However, all have been reported in US feed or feed manufacturing environment (Narayanappa et al., 2015; Ney et al., 2019; Stewart & Jones, 2019). Recently, feed was reported to be a possible vehicle of Senecavirus A transmission in Brazil, but has not been demonstrated in the United States (Leme, Miyabe, Dall Agnol, Alfieri, & Alfieri, 2019). This leads its probability to be characterized as possible, and an overall risk of negligible (Table 4) for feed‐based transmission of these domestic viruses.

While it is concerning that the domestic spread of viruses has been linked to the feed supply chain, the larger looming threat are various foreign animal diseases. These viruses, including foot and mouth disease virus (FMDV), CSFV, ASFV and Chinese pseudorabies virus (PSV) have the potential for entry into the United States through feed or ingredient contamination. Their entry would be detrimental to the naïve domestic swine herd. Preventing foreign animal disease entry is vital to sustaining pork production and subsequent export to international trade partners. Due to the severe consequences of entry, virus transmission through the feed supply chain is a risk worthy of significant investigation and mitigation. Information from here forward will address methods to establish and minimize the probability for viral transmission through feed and feed mills. Their severity is high, and the probability and overall risk of foreign animal disease transmission though a feed vehicle is assessed in the following section.

### 3.2 Factors impacting foreign animal disease transmission in feed

#### 3.2.1 Likelihood of contamination

In order to transmit virus, feed must first come into contact with the pathogen. This may occur at the ingredient stage, where a combination of geographical location, agricultural practices and transportation methods may lead the ingredient to be contaminated. As of November 2018, only 22 of the 182 member countries of the World Organization for Animal Health are free entirely from FMDV, CSFV and ASFV (Dee et al., 2018). While geography is an important factor, the production of the ingredient must also be considered. For example corn cobs dried and stored near roadways or feral pig populations may come into contact with manure or ticks carrying disease prevalent in the region. This type of contamination can occur among a number of international trade partners. However, the same trade partners may have other facilities producing single ingredients in biosecure laboratories. Frequently, amino acids and vitamins are produced in laboratory‐type settings, where this is little risk for contamination except if mixed with contaminated carriers or placed in contaminated packaging or vehicles during distribution. If those biosecure ingredients are transported in single‐use, sealed bags, they would have negligible risk for viral contamination. If transported in re‐used tote bags or dirty containers, they pose a higher risk for pathogen contamination. Unfortunately, there is no direct assessment for safety available, but raw agricultural commodities from countries with circulating virus and ingredients transported in bulk are likely a higher risk for being contaminated with a foreign animal disease compared to products being produced in biosecure facilities and transported in sealed, clean containers. This underscores the risk of post‐processing cross‐contamination. Many products may be produced with a thermal, enzymatic, or pH‐driven step that reduces viral viability. However, processes must be in place to prevent recontamination during packaging or downstream distribution.

#### 3.2.2 Viral survivability

If an ingredient is contaminated with a virus, the pathogen must survive transport to cause infectivity in the US swine herd. For foreign animal diseases, this would involve either trans‐Atlantic or trans‐Pacific shipment in varying temperatures and humidity. The potential for transboundary entry through ingredients has been determined directly for ASFV, and indirectly via surrogates for FMDV, CSFV and PSV (Dee et al., 2018). When subjected to conditions mimicking trans‐Atlantic shipment, ASFV survived in nearly all tested feed ingredients and in stock virus, showing its high survivability. Similarly, the surrogates for FMDV (Senecavirus A) and PSV (swine vesicular disease virus) survived in nearly all ingredients subjected to conditions mimicking trans‐Pacific shipment (Dee et al., 2018). However, the surrogate for CSFV (bovine viral diarrhoea virus) did not survive in any of the tested ingredients, complete feed or stock virus (Dee et al., 2018). While this data is important, it is also limited. First, it is proof‐of‐concept research with high levels of viral inoculum and limited sample size and quantity. Furthermore, the research utilized one combination of time × temperature scenarios. This is problematic, because viral degradation is time × temperature dependent. As temperature fluctuates, virus degrades at varying rates. For example viral degradation would be faster in a hot warehouse during North Carolina summers versus. cold warehouse in the Minnesota winter. Additionally, only 11 ingredients, one complete feed, and stock virus were evaluated. Finally, surrogates were necessary due to the limited number of facilities where this research could be conducted. Future and ongoing efforts must address these same research questions, but in the direct pathogens. Even with these gaps, these data are important in that it establishes the theoretical potential for ingredients to be a transboundary vector of FMDV, ASFV and PSV entr into the domestic feed supply.

#### 3.2.3 Infectivity of the virus

To cause illness within an animal, there must be sufficient quantities of virus within a feed or ingredient to cause infectivity. One of the reasons that PEDV is so easily spread through the feed supply chain is its low infectious dose, just 5.6 × 10^1^ TCID_50_/g in feed has been demonstrated to be infectious via bioassay (Schumacher et al., 2016). This equates to one gram of faeces from an acutely infected pig having the potential to contaminate 500 tonnes of feed with PEDV (Jones, Stark, Dritz, Rigdon, & Woodworth, 2015). The minimum infectious dose of CSFV and FMDV through natural feeding behaviour have yet to be established in feed. However, the infectious dose of ASFV Georgia 2007 in feed was recently been established in swine via swine bioassay (Niederwerder et al., 2019). Importantly, the probability for infection is based on both dose and number of exposures. For example if pigs with a single exposure to feed containing 10^4^ TCID_50_ ASFV have a probability of infection of 25%, the likelihood of infection would increase as the number of exposures increase. The process of feed manufacturing would likely homogenize viral contamination throughout a batch of feed, reducing its dose, but increasing the potential number of exposures. A dose of 10^4^ TCID_50_ across 10 exposures could have an infection probability of nearly 100%. Based on this data, it is possible that even low levels of ASFV may cause infectivity through feed.

#### 3.2.4 Overall risk of feed as a vehicle for foreign swine pathogen transmission

Based on current knowledge of the likelihood of contamination, survivability or infectivity of a pathogen, prevention or mitigation, the probability for FMDV, CSFV and PSV transmission through feed is unlikely, leading to a negligible overall risk (Table 4). If ASFV enters the feed supply chain, viral levels may range widely based on the method of contamination. For example environmental cross‐contamination may be similar to inoculation levels of 10^1^ or 10^2^. However, direct faecal contamination may lead to up to 10^7^. Due to its unpredictability in dose, and that the probability of African swine fever disease virus (ASFV) transmission by feed is possible, leading to a moderate overall risk (Table 4).

As with all models, this risk assessment is liable to change. For example if ASFV enters the United States, its probability of transmission by feed may increase to likely or almost certain, which may increase its overall risk to critical. The US swine and feed industries must continue to increase their knowledge and prevention of biological pathogen transmission in feed so that risk of disease remains negligible to moderate. Furthermore, resources must be directed towards understanding the potential for contamination, survivability and infectivity of *S. enterica* serotype I 4,[5],12:i:‐, PEDV, and ASFV in feed and ingredients. It is necessary to prevent their entry into the feed supply chain and implement science‐based mitigation measures to limit their likelihood of occurrence.

### 3.3 Methods to reduce likelihood of pathogen entry into the feed supply chain

#### 3.3.1 Prevention of entry

If a foreign animal disease contaminates an ingredient in a sufficiently high dose to survive transboundary shipment and cause infectivity, we must prevent its entry into the domestic swine feed supply chain. This is best accomplished by eliminating high‐risk ingredients from mills. Ingredients that are likely to be contaminated based on their combination of geographical location, agricultural practice and transportation methods should be completely eliminated from the entire facility, not just high‐risk diets. This is because once a virus enters a feed manufacturing facility, it tends to spread to surfaces and stay present until sanitation, which is extremely difficult to complete in a feed mill. In a 2017 study, a PEDV‐contaminated ingredient was introduced into a mixer (Schumacher et al., 2017). That process caused nearly all feed contact surfaces (inside of mixer, interior of conveyors, etc.) to have measurable PEDV after the initial diet was manufactured, and the virus stayed present even after four flushes of PEDV‐negative feed batches. Even more concerning, nearly all non‐feed contact surfaces (walls, floors, equipment exteriors, etc.) had measureable virus as soon as PEDV was introduced and remained on these surfaces after all 4 PEDV‐negative feed batches were manufactured. Dust collected from PEDV‐positive, non‐feed‐contact surfaces subsequently caused infectivity in a pig bioassay (Gebhardt et al., 2018). In order to decontaminate the facility, all equipment had to be disassembled and pressure washed with a 10% bleach solution.

It has therefore been established that if an ingredient carries a foreign animal disease into a feed mill, there is potential for the mill itself to become contaminated and become a source of disease transmission. Thus, it is warranted to exclude high‐risk ingredients from the facility altogether. A decision tree has been developed by the Swine Health Information Center to help facilities identify high‐risk ingredients (Swine Health Information Center, 2018). Common high‐risk ingredients to exclude from mills include soybean‐based ingredients from countries with circulating foreign animal disease, microingredients that contain vegetable‐based carriers sourced from countries with circulating foreign animal disease, and porcine‐derived ingredients.

While ingredients are one method of pathogen entry into the mill, the feed manufacturing facility can be contaminated by other vectors, such as people and vehicles. For these reasons, biosecurity practices common on farms should be extended to feed mills to limit the potential of pathogen transmission through the feed supply chain. Mats should be placed over receiving pits, delivery vehicles routed to avoid crossing with ingredient trucks, and lines of separation implemented to control personnel foot traffic. In times of high pathogen risk, facilities should consider sanitizing trucks prior to entering receiving or load‐out bays. A biosecurity plan can be a useful tool to identify possible points of pathogen entry (Cochrane et al., 2016).

To determine gaps in biosecurity, facilities should consider proactive surveillance. Analysing environmental or product samples for viruses is expensive, and sometimes impossible in the case of foreign animal diseases. Instead, facilities may consider analysing environmental samples for Enterobacteriaceae, a family of bacteria that includes both pathogenic (*Salmonella*, *Escherichia coli*) and non‐pathogenic genera. Enterobacteriaceae has been successfully used as indicator of facility hygiene in human food, pet food and rendering facilities (Schothorst & Oosterom, 1984; Stewart & Jones, 2019). Analysis is rapid and inexpensive, and generally a good indicator for porcine virus presence. Previous research has confirmed Enterobacteriaceae presence is correlated with PEDV, porcine deltacoronavirus and Senecavirus A presence in feed mills (Sardella et al., 2019; Stewart & Jones, 2019). This type of environmental monitoring can establish baseline Enterobacteriaceae levels in facilities, and identify poor hygiene areas that are likely to be risk points for pathogen entry. In a study of US swine feed mills, worker shoes and receiving pits contained the greatest prevalence of Enterobacteriaceae (Magossi et al., 2019). To best prevent pathogen entry into the feed supply chain, facilities must exclude high‐risk ingredients and maximize biosecurity to prevent transmission via ingredients, people and vehicles.

#### 3.3.2 Mitigation of pathogens in feed

Even with the best efforts to prevent foreign animal disease entry into a feed mill, there is still the potential for its presence and subsequent transmission through feed. As a final hurdle to prevent transmission to pigs, facilities may consider proactive mitigation through quarantining ingredients, thermal processing or the use of feed additives. Quarantining ingredients to allow for natural viral degradation may be an effective method of mitigation; however, there is limited information to carry it out successfully. The concept of viral decay can be used to calculate half‐life estimates for quarantine time recommendations (Dee et al., 2014). Unfortunately, currently available data are based only on two data points (d 0 and 30) with one time × temperature combination. More robust thermal decay curves are needed to more accurately estimate quarantine times across a broader range of environmental conditions before the mitigation can be used confidently.

Another mitigation method, thermal processing, has had demonstrated success to reduce the infectivity of PEDV in feed (Cochrane et al., 2017). Again, the method's success relies on time × temperature combinations that have not yet been fully established. In the case of ASFV, pelleting is not a plausible mitigation measure because thermal decay curves involving temperatures achieved through a traditional steam conditioner have never been established. Furthermore, quarantine time and thermal processing are both considered point‐in‐time mitigation measures. Both may, under ideal conditions, lead to viral inactivation. However, neither protect the ingredient nor feed from subsequent downstream cross‐contamination that may occur during conveyance, load‐out or transportation.

Due to the potential for cross‐contamination, feed additives may be more successful mitigants. Formaldehyde‐based ingredients or those containing medium chain fatty acids have had demonstrated success as mitigants of porcine pathogens (Gebhardt et al., 2018). Their potential is still being evaluated in mitigating foreign animal diseases in feed and ingredients. Still, these ingredients must be used safely and in compliance with regulatory requirements. For example formaldehyde is an approved food additive for the prevention of *Salmonella* in feed, but its use for PEDV or ASFV control would be outside of the current regulatory approval. As research continues to identify products and additives to successfully mitigate pathogens, it will be a key to maintain dialogue with regulatory agencies so the products can be used legally and safely.

In conclusion, there are two domestic porcine pathogens (*S. enterica* serotype I 4,[5],12:i:‐ and PEDV) and one foreign animal disease (ASFV) that pose the greatest risk for entry and transmission through a feed vehicle in the United States. Additional research is urgently needed to fully assess the probability of their occurrence, as well as methods to reduce their likelihood of entry or potential mitigation.

## ETHICS STATEMENT

The authors confirm that the ethical policies of the journal, as noted on the journal's author guidelines page, have been adhered to. No ethical approval was required as this is a review article with no original research data.

## CONFLICT OF INTEREST

The authors have no conflicts of interest to declare.
